# Nuclear Expression of GS28 Protein: A Novel Biomarker that Predicts Worse Prognosis in Cervical Cancers

**DOI:** 10.1371/journal.pone.0162623

**Published:** 2016-09-09

**Authors:** Uiju Cho, Hae-Mi Kim, Hong Sik Park, Oh-Joo Kwon, Ahwon Lee, Seong-Whan Jeong

**Affiliations:** 1 Department of Hospital Pathology, St. Vincent’s Hospital, College of Medicine, The Catholic University of Korea, Suwon, Republic of Korea; 2 Department of Biochemistry, College of Medicine, The Catholic University of Korea, Seoul, Republic of Korea; 3 Department of Hospital Pathology, Seoul St. Mary’s Hospital, College of Medicine, The Catholic University of Korea, Seoul, Republic of Korea; Institut national de la recherche scientifique, CANADA

## Abstract

**Objective:**

The protein GS28 (28-kDa Golgi SNARE protein) has been described as a SNARE (Soluble N-ethylmaleimide-sensitive factor attachment protein receptors) protein family member that plays a critical role in mammalian ER-Golgi or intra-Golgi vesicle transport. Little is known about the possible roles of GS28 in pathological conditions. The purpose of this study was to evaluate GS28 expression in cervical cancer tissues and explore its correlation with clinicopathological features and prognosis.

**Methods:**

We investigated GS28 expression in 177 cervical cancer tissues by using immunohistochemistry and evaluated the correlation of GS28 expression with clinicopathological features, the expression of p53 and Bcl-2, and prognosis of cervical cancer patients. Immunoblotting was performed using six freshly frozen cervical cancer tissues to confirm the subcellular localization of GS28.

**Results:**

Immunoreactivity of GS28 was observed in both nuclear and cytoplasmic compartments of cervical cancer cells. High nuclear expression of GS28 was associated with advanced tumor stages (P = 0.036) and negative expression of p53 (P = 0.036). In multivariate analyses, patients with high nuclear expression of GS28 showed significantly worse overall survival (OS) (hazard ratio = 3.785, P = 0.003) and progression-free survival (PFS) (hazard ratio = 3.019, P = 0.008), compared to those with low or no nuclear expression. It was also a reliable, independent prognostic marker in subgroups of patients with early stage T1 and negative lymph node metastasis in OS (P = 0.008 and 0.019, respectively). The nuclear expression of GS28 was confirmed by immunoblotting.

**Conclusion:**

High nuclear expression of GS28 is associated with poor prognosis in early-stage cervical cancer patients. GS28 might be a novel prognostic marker and a potential therapeutic target in cervical cancer treatment.

## Introduction

Cervical cancer is the third most prevalent malignancy and the fourth leading cause of cancer deaths among women worldwide, despite the successful Papanicolaou smear based screening and treatment program [1]. Although patients with early-stage cervical cancer have good prognoses with five-year survival rates of 90–95%, a significant number of patients die due to relapses [2]. Tumor stage and size, presence of lymphovascular invasion, lymph node metastasis, and remnant tumors in resection margins are powerful markers of aggressive disease; however, they do not fully account for the observed variability in patient outcomes. Patients with a high risk of recurrence following surgery receive adjuvant radiotherapy, with or without chemotherapy. Cisplatin is commonly used for the treatment of cervical cancer; however, drug resistance is common and serious side effects may develop [3]. Therefore, more effective and safe approaches are required to tackle the survival of some cancer cells that cause the failure of treatment. Moreover, the identification of biomarkers to predict the potential progression of cancer and prognosis of patients with cervical cancer is desirable for planning appropriate individualized therapies.

The Golgi apparatus functions as a factory in which membrane transport intermediates received from the endoplasmic reticulum (ER) are processed further and sorted for delivery to their eventual destinations–lysosomes, plasma membrane, or secretion [4]. Soluble N-ethylmaleimide-sensitive factor attachment protein receptors (SNAREs) are a group of tail-anchored membrane proteins that play important roles in the membrane trafficking steps. In mammalian cells, at least 12 different proteins composing SNARE have been identified in the Golgi apparatus [5]. Moreover, the Golgi apparatus has been demonstrated to be a platform for molecular signaling between Golgi and other organelles [6]. Through organelle networking, the Golgi apparatus is involved in crucial cellular activities, including stress sensing/effecting, cell death, mitosis checkpoints, and malignant transformation [6]. Numerous proapoptotic/autophagic factors and mitosis-related molecules are localized to the Golgi [7]. Therefore, Golgi is becoming increasingly important as an anti-cancer target.

GS28 (Golgi SNARE protein, 28 kDa) has been described as a member of the SNARE protein family that plays a critical role in ER—Golgi or intra—Golgi vesicle transport [8,9]. Until now, all studies have focused on the function of GS28 in vesicular transport, and little is known about its potential roles in pathological conditions. A recent study demonstrated that deletion mutants of GS28 in *Caenorhabditis elegans* show reduced seam cell numbers and a missing ray phenotype during development, suggesting that GS28 has roles in cell proliferation and differentiation [10]. Mutations in GS28 also lead to retinal degeneration in *Drosophila* [11]. However, there has been no research yet on the role of GS28 protein in human cancer tissues.

In this study, we evaluated GS28 expression for its potential clinical use in cervical cancer treatment. This is the first study to assess the prognostic value of GS28 in cervical cancers.

## Materials and Methods

### Patients and tissue samples

We collected 177 archival cases of cervical cancer (139 squamous cell carcinomas, 27 adenocarcinomas, and 11 adenosquamous cell carcinomas) between the years of 1999 and 2002, from the archives of the Department of Pathology at the Seoul St. Mary’s Hospital. All the patients underwent surgical resection and were treated according to standard treatment guidelines, as outlined during that timeframe, regarding chemotherapy and radiotherapy. The original hematoxylin and eosin (H&E)-stained sections were reviewed by two expert pathologists (AL and UC). Diagnoses and pathologic data were confirmed by histological review. Patient demographics and clinical data were analyzed retrospectively, using the hospital medical records. Pathologic stages were categorized according to the seventh edition of the TNM classification by the American Joint Committee on Cancer and the Revised International Federation of Gynecology and Obstetrics (FIGO) staging systems [12]. According to the clinical characteristics, tumors in stage T1 were defined as early cervical cancer and those in stages T2, T3, and T4 were defined as advanced cancer. Survival data were obtained from the medical records. In addition, we retrieved freshly frozen tissues of six cases of cervical cancer, from the tissue bank at the Seoul St. Mary’s hospital. All samples and medical record data were anonymized before use in this study and the participants did not provide written informed consent. The use of medical record data and tissue samples for this study was approved by the Institutional Review Board of Seoul St. Mary’s hospital and the Catholic University of Korea (KC15SISI0475, MC12SISI0168).

### Tissue microarray construction

We constructed tissue microarrays (TMAs) from 177 formalin-fixed paraffin-embedded (FFPE) cervical cancer blocks. Based on the H&E-stained sections, a morphologically representative tumor area in each of the donor blocks was selected. Each donor block was cored, and a 2-mm core was transferred to the recipient paraffin block, using a tissue micro arrayer device (Micro Digital Co., Gunpo-si, Gyeonggi-do, Korea). Each recipient block contained 30 cases of cervical cancer. One slide from each of the TMA blocks was stained with H&E, to confirm the presence of tumor tissue.

### Immunohistochemical analysis

For immunohistochemical (IHC) analyses of GS28, p53, and Bcl-2, the TMA blocks were sectioned at 4-μm thickness and mounted on precoated glass slides. The slides were deparaffinized in xylene and endogenous peroxidase activity was blocked, for staining. IHC assays for GS28 (1/GS28, mouse monoclonal, 1:1000; BD Biosciences, San Jose, CA, USA), p53 (DO-7, mouse monoclonal, 1:100; Dako, Glostrup, Denmark), and Bcl-2 (124, mouse monoclonal, 1:50; Dako, Glostrup, Denmark) were performed using Ventana NX automated IHC system (Ventana Medical Systems, Tucson, Arizona, USA). Heat-induced antigen retrieval was performed using Cell Conditioning solution (CC1, a proprietary buffer). Immunostainings were visualized using diaminobenzidine as the chromogen, with normal cervical tissues serving as positive controls, for all the antibodies tested.

Immunohistochemical staining was assessed by two pathologists (A.L. and U.C), in a blinded manner. In the case of disagreement, the staining was reviewed together by both the observers to obtain a consensus score. For a semiquantitative assessment of nuclear and cytoplasmic GS28 expression, the histochemical score (H-score) was used. The intensity score was assigned as follows: score 3, strong staining that is definitely more intense than the staining of normal cervical epithelium; score 2, moderate staining of the tumor cells; score 1, faint or similar intensity to the staining in the normal cervical epithelium; and score 0, none of the above. In addition, the percentage of positive cells at each intensity was evaluated. The H-score was calculated as 1 × % of cells stained at score 1 + 2 × % of cells stained at score 2 + 3 × % of cells stained at score 3. For analytical purpose, H-scores were categorized into either “low-” or “high-” expression level based on the cut-off value generated by receiver operating characteristic (ROC) curve analysis. p53 and Bcl-2 expression were scored as positive if the nuclei were stained in ≥ 10% of the tumor cells.

### Preparation of tissue extracts and immunoblotting

To verify the expression of GS28, tissue extracts were prepared from six freshly frozen tissues of cervical cancer. Whole, cytoplasmic, and nuclear extracts were prepared using NE-PER Nuclear and Cytoplasmic Extraction Reagents (Thermo Fischer Scientific, Rockford, IL, USA), according to the manufacturer’s instructions. Equal proportions of each fractionated extract were loaded and separated by SDS-polyacrylamide gel electrophoresis, and the proteins were transferred onto polyvinylidene difluoride membranes. The membranes were incubated with GS28 antibody (1/GS28, mouse monoclonal; BD Bioscience) and then with secondary antibodies conjugated to horseradish peroxidase. Immunoreactive protein signals were visualized using ECL^TM^ Western Blotting Detection Reagents (GE Healthcare, Buckinghamshire, England). Laminin B1 and α-tubulin were used as the nuclear and cytoplasmic control markers, respectively.

### Statistical analysis

Overall survival (OS) time was measured from the time of initial diagnosis until death, or until the end of follow-up. Progression-free survival (PFS) time was measured from the time of initial diagnosis until disease recurrence or metastasis. Survival data were analyzed using Kaplan-Meier survival curves and the differences between curves were analyzed using log-rank tests. Multivariate analysis for the OS and PFS were performed using the Cox proportional hazards model. Two-tailed p-values <0.05 were considered significant. The χ^2^ test or Fisher’s exact test was used to analyze the correlation between GS28 expression and clinicopathologic parameters, and Mann-Whitney U test was used to analyze the differences in median values. Analysis and data graphing listed above were performed using SPSS 21.0 (IBM, Armonk, New York, USA). Additionally, the specificity and sensitivity for the outcome were plotted to generate a receiver operating characteristic (ROC) curve. A score closest to the point with both maximum specificity and sensitivity was selected as a prognostically relevant cut-off score leading to the largest group of tumors correctly classified as having or not having the clinical outcome (overall survival). Generation and analysis of the ROC curve were performed using MedCalc statistical software package 16.4.3 (MedCalc Software, Ostend, Belgium).

## Results

### Patient characteristics

The median age of the 177 cervical cancer patients included in this study was 49.6 years (range, 23–75 years). 78.5% of the patients had squamous cell carcinoma, 15.3% had adenocarcinoma, and 6.2% had adenosquamous carcinoma. At the time of diagnosis, 82.5% of the patients had moderately differentiated tumors; 68.4% and 30.5% had pathologic stage T1 and T2 cancer, respectively; and 25.4% had lymph node metastases. Per the FIGO staging system, 57.1% of the patients were in stage 1, 18.1% in stage 2, 23.7% in stage 3, and 1.1% in stage 4 (Table 1).

**Table 1:** Relationship of subcellular expression of GS28 with clinicopathological features of cervical cancer tissues.

	All patients (*N* = 177)	Nuclear GS28 expression			Cytoplasmic GS28 expression		
		Low (*N* = 126)	High (*N* = 51)	*P*	Low (*N* = 60)	High (*N* = 117)	*P*
Age, median (range)	49.6 (10.6)	48 (23–75)	51 (25–72)	0.051^†^	47 (23–64)	50 (25–75)	0.580^†^
Histologic diagnosis							
Squamous cell carcinoma	139 (78.5)	95 (75.4)	44 (86.3)	0.208	49 (81.7)	90 (76.9)	0.514
Adenocarcinoma	27 (15.3)	23 (18.3)	4 (7.8)		9 (15.0)	18 (15.4)	
Adenosquamous carcinoma	11 (6.2)	8 (6.3)	3 (5.9)		2 (3.3)	9 (7.7)	
Histologic differentiation							
Well	11 (6.2)	10 (7.9)	1 (2.0)	0.329	4 (6.7)	7 (6.0)	0.056
Moderate	146 (82.5)	102 (81.0)	44 (86.3)		54 (90)	92 (78.6)	
Poor	20 (11.3)	14 (11.1)	6 (11.8)		2 (3.3)	18 (15.4)	
> 1cm depth of invasion into cervical wall	96 (54.2)	66 (52.4)	30 (58.8)	0.625	31 (51.7)	65 (55.6)	0.623
Lymphovascular invasion	95 (53.7)	65 (51.6)	30 (58.8)	0.382	39 (65.0)	56 (47.9)	**0.030**
Perineural invasion	18 (10.2)	12 (9.5)	6 (11.8)	0.655	4 (6.7)	14 (12.0)	0.270
Endometrial invasion	27 (15.3)	17 (13.5)	10 (19.6)	0.305	10 (16.7)	17 (14.5)	0.708
Parametrial invasion	43 (24.3)	28 (22.2)	15 (29.4)	0.312	17 (28.3)	26 (22.2)	0.370
pT1 stage	121 (68.4)	92 (73.0)	29 (56.9)	**0.036**	39 (65.0)	82 (70.1)	0.491
FIGO stage I	101 (57.1)	78 (61.9)	23 (45.1)	**0.041**	34 (56.7)	67 (57.3)	0.939
Lymph node metastasis	45 (25.4)	29 (23.0)	16 (31.4)	0.248	13 (21.7)	32 (27.4)	0.411

SD, standard deviation.

All the parameters are presented in number (%), except for age; Bold value indicates P < 0.05.

^†^Mann-Whitney U test was used for the intergroup mean comparison.

### Evaluation of GS28 expression and its association with clinicopathological features

**Fig 1:**
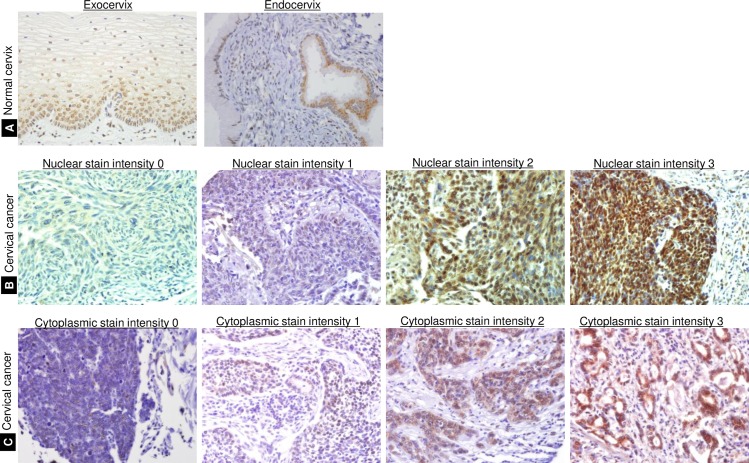
GS28 immunohistochemical staining in the cervix. (A) In normal cervical tissues, GS28 immunostaining is distributed in the cytoplasmic and nuclear compartments of exocervical squamous cells and endocervical glandular cells. The staining intensity varies, but it is mostly weak. (B) In cervical cancer tissues, the staining intensity of GS28 varies widely from no staining (score 0) or weak staining (score 1) to moderate or strong staining (scores 2 and 3), in the nuclear compartment. (C) GS28 expression is also seen in the cytoplasmic compartments of cervical cancer tissues with varying intensities, from scores 0 to 3.

In the epithelium of control tissues (normal cervix), immunostaining of GS28 was weak either in the nucleus or cytoplasm or both (Fig 1). In many cervical cancer cases, GS28 showed diffuse homogeneous staining pattern but there were differences in the location and the intensity of the staining (cytoplasm vs. nucleus) in each cells. When nuclear and cytoplasmic staining were scored separately, mean H-scores were 84.7 (standard deviation 86.5) and 98.1 (standard deviation 84.4), respectively. ROC curve analysis indicated that H-score 150 to be the best cut-off value for nuclear expression of GS28 for predicting the overall survival (area under the curve = 0.641). The sensitivity of this cut-off value was 54.17 (95% CI, 32.8–74.4) and the specificity was 75.82% (95% CI, 68.2–82.4). For cytoplasmic expression of GS28, the best cut-off vale was 30 for predicting the overall survival (area under the curve = 0.557) with the sensitivity 45.83 (95% CI, 25.6–67.2) and the specificity 67.97 (95% CI, 60.0–75.3). Using these cut-offs, 51 (H-score >150, 28.8%) and 117 (H-score >30, 66.1%) cases showed high immunoreactivity in the nuclear and cytoplasmic compartments, respectively. High nuclear expression of GS28 was correlated with higher T stages (P = 0.036) and FIGO stages (P = 0.041); however, high cytoplasmic expression of GS28 did not correlate with the T stage (Table 1). Instead, high cytoplasmic expression was correlated with the presence of lymphovascular invasion (P = 0.03). Other clinicopathologic features, including age, histologic diagnosis, histologic differentiation, depth of invasion, perineural invasion, endometrial invasion, parametrial invasion, and lymph node metastasis showed no correlation with the expression of GS28 (P > 0.05) (Table 1).

### Association of GS28 expression with p53 and Bcl-2 protein

As GS28 has been implicated to play a role in the mammalian apoptotic process [13], we evaluated the correlation between the expression of GS28, the tumor suppressor, p53, and the anti-apoptotic protein, Bcl-2. The expression of p53 and Bcl-2 protein was seen in 34% (70/177) and 24.3% (43/177), respectively, of all the cancer tissues examined. High nuclear expression of GS28 showed a statistically significant inverse correlation with p53 expression (P = 0.036) and high cytoplasmic expression of GS28 was also inversely correlated with Bcl-2 expression (P < 0.001) (Table 2).

**Table 2:** Relationship of nuclear expression of GS28 with p53 and Bcl-2 expression in cervical cancer tissues.

Parameters	Nuclear GS28 expression		*P*	Cytoplasmic GS28 expression		*P*
	Low	High		Low	High	
p53, no. (%)						
Negative	70 (55.6)	37 (72.5)	**0.036**	35 (58.3)	72 (61.5)	0.68
Positive	56 (44.4)	14 (27.5)		25 (41.7)	45 (38.5)	
Bcl-2, no. (%)						
Negative	98 (77.8)	36 (70.6)	0.312	56 (93.3)	78 (66.7)	**<0.001**
Positive	28 (22.2)	15 (29.4)		4 (6.7)	39 (33.3)	

Bold value indicates P < 0.05.

### Nuclear GS28 expression and prognostic prediction in cervical cancer patients

**Fig 2:**
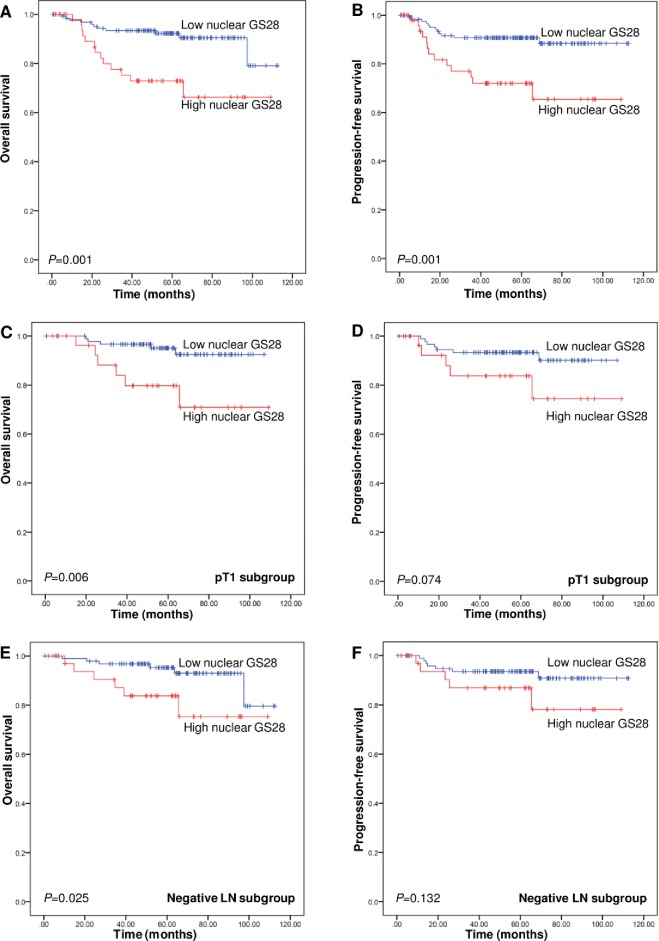
Kaplan-Meier curves of univariate analysis data (log-rank test). (A) The overall survival (OS) and (B) progression-free survival (PFS) of cervical cancer patients with high versus low nuclear expression of GS28. (C) The OS and (D) PFS of patients with stage T1 tumors (pT1 subgroup), and (E) the OS and (F) PFS of patients without lymph node metastasis (negative-lymph node-metastasis subgroup) with high versus low nuclear expression of GS28.

After a median follow-up duration of 56.1 (range, 0.5–112.5) months, the five-year OS and PFS rates were 87% and 86%, respectively. We carried out univariate survival analysis with respect to the subcellular expression pattern of GS28 (nuclear or cytoplasmic localization), clinicopathologic features, and p53 and Bcl-2 expression, in the patients. We found that the following clinicopathologic features were associated with worse OS and PFS, based on the univariate analysis: lymphovascular invasion, perineural invasion, parametrial invasion, T stage, FIGO stage, and lymph node metastasis (P < 0.05). Poor histologic differentiation was associated with worse PFS (P = 0.013). The expression of p53 and Bcl-2 were not significant prognostic factors (P = 0.428 and 0.44 in OS, and P = 0.844 and 0.333 in PFS, respectively). Univariate analysis indicates that cervical cancer patients with high nuclear expression of GS28 had significantly shorter OS and PFS than patients with low nuclear GS28 expression (mean OS, 82.7 vs. 102.8 months; log-rank test, P = 0.001; and mean PFS, 80.8 vs. 102.5 months; log-rank test, P = 0.001) (Fig 2A and 2B). However, patients with high expression of cytoplasmic GS28 expression demonstrated no difference in OS (log-rank test, P = 0.351) and PFS compared to low expression group (log-rank test, P = 0.876).

Using multivariate analysis, high nuclear GS28 expression was identified to be an independent predictor of OS (hazard ratio = 3.785, P = 0.003) and PFS (hazard ratio = 3.019, P = 0.008) (Table 3). In addition to high nuclear GS28 expression, perineural invasion, parametrial invasion and T stage were also identified to be the independent prognostic parameters in OS (Table 3).

**Table 3:** Multivariate Cox regression analysis for overall survival and progression-free survival in cervical cancer patients (N = 177).

Parameters	Overall survival		Progression free survival	
	Hazard ratio (95% CI)	*P*	Hazard ratio (95% CI)	*P*
High nuclear GS28 expression	3.785 (1.569–9.131)	**0.003**	3.019 (1.333–6.836)	**0.008**
Lymphovascular invasion	1.551 (0.520–4.627)	0.431	1.880 (0.656–5.388)	0.240
Perineural invasion	2.921 (1.094–7.798)	**0.032**	2.849 (1.100–7.376)	**0.031**
Parametrial invasion	7.426 (1.356–40.68)	**0.021**	2.518 (0.697–9.100)	0.159
T stage (reference: stage 1)		**0.001**		0.999
Stage 2	0.367 (0.068–1.977)	0.244	0.966 (0.268–3.479)	0.966
Stage 3, 4	43.85 (0.748–918.1)	0.015	0.001 (0—NR)	0.990
Lymph node metastasis	2.140 (0.748–6.123)	0.156	2.174 (0.846–5.586)	0.107

NR, not reached; Bold values indicate *P* < 0.05.

We further evaluated the prognostic value of high nuclear expression of GS28 in specific subgroups of patients, who were stratified according to the T stage and lymph node metastasis. Of the 121 stage T1 cancers, 29 cases showed high nuclear expression of GS28 and were associated with worse OS (mean OS, 88.8 vs. 102.1 months; log-rank test, P = 0.006) (Fig 2C) but not with PFS (mean PFS, 90.2 vs. 99.7 months, log-rank test, P = 0.074) (Fig 2D), according to univariate analysis. Moreover, multivariate analysis showed that the high nuclear expression of GS28 in T1 cervical cancers is a poor independent prognostic factor of OS (hazard ratio = 5.252, P = 0.008) (Table 4). In advanced T stage group, the high nuclear expression of GS28 showed no prognostic value in OS and PFS (log rank, all P > 0.05).

**Table 4:** Multivariate Cox regression analysis for overall survival and progression-free survival in stage T1 subgroup (N = 121) of cervical cancer patients.

Parameters	Overall survival		Progression free survival	
	Hazard ratio (95% CI)	*P*	Hazard ratio (95% CI)	*P*
High nuclear GS28 expression	5.252 (1.555–17.743)	**0.008**	3.014 (0.944–9.618)	0.062
Lymphovascular invasion	1.222 (0.299–4.989)	0.780	1.817 (0.529–6.243)	0.343
Perineural invasion	3.571 (0.934–13.657)	0.063	3.319 (0.890–12.383)	0.074
Lymph node metastasis	2.150 (0.446–10.361)	0.340	0.857 (0.167–4.387)	0.857

Bold values indicate *P* < 0.05.

The high nuclear expression of GS28 was also strongly associated with worse OS (mean OS, 91.7 vs. 105.5 months; log-rank test, P = 0.025) in patients without lymph node metastasis at the time of the surgery (Fig 2E) but it was not associated with PFS (mean PFS, 93.2 vs. 105.1 months; log-rank test, P = 0.132) (Fig 2F) in this group. Hence, multivariate analysis revealed that high nuclear expression of GS28 is an independent prognostic factor of OS (hazard ratio = 3.969, P = 0.019) for patients without lymph node metastasis but not for patients with lymph node metastasis (P = 0.298) (Table 5).

**Table 5:** Multivariate Cox regression analysis for overall survival and progression-free survival in negative-lymph node-metastasis subgroup (N = 132) of cervical cancer patients.

Parameters	Overall survival		Progression-free survival	
	Hazard ratio (95% CI)	*P*	Hazard ratio (95% CI)	*P*
High nuclear expression of GS28	3.969 (1.249–12.607)	**0.019**	2.745 (0.859–8.774)	0.088
Lymphovascular invasion	2.000 (0.621–6.443)	0.246	3.103 (0.924–10.428)	0.067
Perineural invasion	5.490 (1.346–22.385)	**0.018**	3.646 (0.954–13.936)	0.059
Parametrial invasion	2.311 (0.569–9.393)	0.242	0.626 (0.078–5.024)	0.660

Bold values indicate P < 0.05.

### Subcellular distribution of GS28 protein in tissue extracts

**Fig 3:**
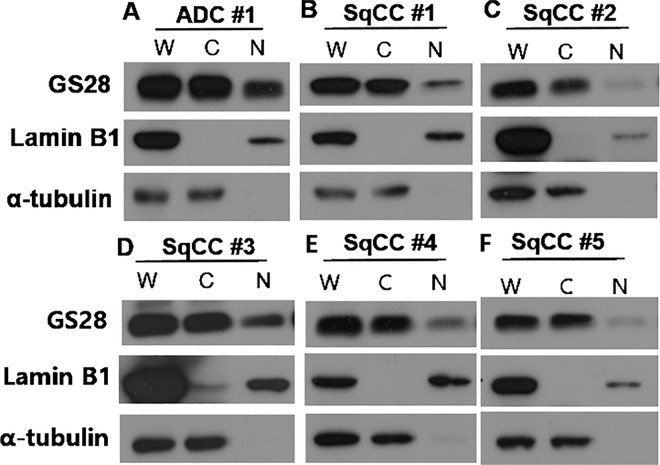
Immunoblotting of cervical cancer tissue extracts. Whole (W), cytoplasmic (C), and nuclear (N) fractions were prepared from tissues of one adenocarcinoma (ADC) and five squamous cell carcinomas (SqCC), and GS28 expression in the fractions was examined by immunoblotting using the anti-GS28 antibody. Laminin B1 and α-tubulin were used as the nuclear and cytoplasmic markers, respectively.

The unexpected and distinct nuclear localization of GS28 protein prompted us to verify its subcellular distribution by immunoblotting (Fig 3). Efficient fractionation was confirmed by immunodetection of α-tubulin and lamin B1, which are localized only in the cytoplasm and nucleus, respectively. We confirmed the presence of significant amounts of GS28 protein in nuclear fractions from four out of the six freshly frozen cervical cancer tissues examined. The nuclear localization of GS28 protein is consistent with our findings based on the immunohistochemical analysis described above.

## Discussion

In this study, we found that high nuclear expression of GS28 in the primary cervical cancer tissues showed significant correlation with higher T stages, and poor PFS and OS. Moreover, multivariate analyses revealed that high nuclear expression of GS28 is a poor independent prognostic factor in cervical cancer patients. When stratified into subgroups, the high nuclear expression was found to be associated with poor OS in stage T1 subgroup and negative lymph node metastasis. Furthermore, we confirmed the significant inverse correlation of nuclear GS28 expression with p53 expression, and demonstrated the nuclear localization of GS28 protein in cervical cancer tissue by immunoblotting. This is the first study to examine GS28 protein expression in human cancer tissues and find a significant correlation of GS28 expression with clinicopathological parameters in cervical cancer.

The endoplasmic reticulum (ER) and Golgi apparatus are two major organelles that play important roles in the processing, sorting, and transporting newly synthesized secretory and transmembrane proteins [7]. ER-Golgi network is also a hub for various signaling pathways that are involved in crucial cellular activities, including cell death and malignant transformation [6]. Novel functions of proteins localized in Golgi have been discovered recently **−** caspase-2 in early apoptotic events [14], polo-like kinase 3 in mitosis and apoptosis [15], and GD3 in triggering mitochondrial membrane permeability [16]. Therefore, the interest in Golgi—the forgotten organelle **−** as an anti-cancer target has been gaining momentum.

SNARE proteins play an essential role in membrane fusion in virtually all eukaryotic cells and thus mediate diverse cellular processes, including synaptic transmission, intracellular trafficking of proteins and lipids, hormone signaling, cell growth and cell migration [17]. Aside from their physiological functions, a number of SNAREs, such as syntaxin 1, have been found to play important roles in tumor cell survival and proliferation in *in vitro* studies [17–19]. Moreover, Lu et al suggested that the SNARE proteins, SNAP-25 and syntaxin, are tumor-specific proteins in parathyroid tissues since their expression is restricted to pathological parathyroid lesions [20].

Among SNARE proteins, GS28 is a membrane protein that is known to play an essential role in intra-Golgi or ER–Golgi vesicle transport [8]. There have been only a few studies on the role of GS28 protein in pathological conditions, besides its roles in vesicular transport. Studies on GS28 mutants in *C*. *elegans* and *Drosophila* suggest that GS28 plays important roles in the proliferation and differentiation of seam cells and the maintenance of retinal neurons [10,11]. Sun et al [13] revealed that GS28 stabilizes p53 through the inhibition of its ubiquitin ligase, MDM2, by binding to it in a cisplatin-dependent manner. Although their findings offered the first evidence that GS28 can be involved in chemosensitivity, these results are only applicable to *in vitro* studies on cell lines. We had reported previously that GS28 plays a protective role in hydrogen peroxide-induced cell death via the inhibition of p38 MAPK in glutathione-depleted neuronal cells [21]. However, so far there has been no research on the expression of GS28 protein in human pathological tissues.

We predicted the conserved motifs of the GS28 protein (250 amino acids) using the web-based software, PROSITE and PredictProtein. The predictions displayed only one hit—coiled coil helices (called the SNARE motif) that mediate the interactions between SNARE proteins. The GS28 protein does not contain the nuclear localization signal motif. The motifs with a high probability of occurrence are glycosylation sites and target sites of phosphorylation by casein kinase II (CKII), protein kinase C (PKC), and cAMP- and cGMP-dependent protein kinases. Overexpression of nuclear CK II was shown to be a poor prognostic factor in colorectal cancer [22]. However, the phosphorylation of GS28 and its nuclear localization have not been reported yet. Further studies should confirm the molecular mechanisms of protein kinases and GS28 phosphorylation in cervical cancers.

As GS28 is present throughout the Golgi apparatus, its subcellular location has been expected to be restricted to the cytoplasmic compartment [13] [23] [24]. However, from our immunohistochemical and immunoblot analyses, it is clear that GS28 protein is expressed not only in the cytoplasmic compartment but also in the nuclear compartment of cervical tumor cells. Syntaxin 17, another SNARE protein, is expressed in the cytoplasm and/or nucleus in different tissues, which raises the possibility that the protein may have additional functions in cell proliferation or transformation, besides membrane trafficking [25]. The mechanism underlying the nuclear expression of the SNARE proteins, GS28 and syntaxin 17, is unknown. The mechanism underlying the nuclear expression of the SNARE proteins, GS28 and syntaxin 17, is unknown. It would be of interest whether GS28 partner proteins–such as syntaxin 5, Ykt6, GS15 [5]–are coupled with the GS28 transport from cytoplasmic to nuclear compartment of the tumor cells or not. Further studies should be done to elucidate the molecular mechanisms of the GS28 transport.

A group of SNARE proteins was found to promote tumorigenesis and metastasis [17]. Studies reported the high expressions of SNARE proteins in several types of cancers. Knockdown of α-SNAP protein induced epithelial cell apoptosis via down-regulation of Bcl-2 [26], but no more study is reported on interactions of SNARE proteins and anti-apoptotic protein Bcl-2. In our study, increased expression of cytoplasmic GS28 was correlated with decreased Bcl-2 protein. The significance of the finding is not explainable because expression of cytoplasmic GS28 is not correlated with prognostic parameters.

p53 is a nuclear phosphoprotein that inhibits cell cycle progression and induces apoptosis by regulating several target genes. Based on *in vitro* studies, Sun et al concluded that GS28 may play a positive role in cisplatin-dependent apoptosis by regulating the stability, apoptotic activity, and chemosensitivity of p53 [13]. Our results also demonstrate that the nuclear expression of GS28 is inversely correlated with p53 expression. Although several researchers have investigated whether p53 protein expression in cervical cancers might be a prognostic marker, the results were inconsistent [27]. p53 is more commonly inactivated by E6 protein of human papilloma virus and its decreased function results in proliferation of cells that are prone to acquire additional mutations that may lead to cervical cancer development [28]. However, we have not determined the expression levels of p53 and types of p53 mutations in the cervical cancer tissues analyzed. Collectively, interactions between GS28 and p53 in cervical cancer cells might be involved in different mechanisms than those expected. Our findings may provide new insights into the regulation of p53 in cervical cancer, though further investigations are needed.

Our results demonstrate that high nuclear expression of GS28 protein can be an independent prognostic marker for cervical cancer. To our knowledge, no clinical data that deals with the prognostic value of GS28 in human cancer has been reported yet. Cervical cancer patients who have stage T1 tumors undergo surgery alone or receive adjuvant radiotherapy with/without concurrent cisplatin-based chemotherapy if there is a combination of high-risk factors (i.e., positive pelvic node metastasis, positive surgical margin, and lymphovascular invasion). Although powerful, these factors do not fully account for the variable outcomes of the patients. The significant association of high nuclear expression of GS28 with shorter OS in stage T1 and in the negative lymph node metastasis subgroup indicate that GS28 expression pattern might be useful to effectively identify high-risk patients for adjuvant therapy, thus avoiding other unnecessary therapies. The functional consequence of GS28 accumulation in the nucleus and the exact mechanism by which it influences the clinical outcome in cervical cancers is yet to be investigated, and these are the subjects of our ongoing research.

Notably, we have discovered a novel, powerful prognostic marker for cervical cancer patients, especially those with early-stage cancers at a time when there are only a few useful biomarkers for predicting clinical outcomes [29].

In conclusion, we have shown for the first time the expression of GS28 and its nuclear localization in cervical cancers, and demonstrated that GS28 is a novel prognostic marker of early cervical cancer. Our results suggest that GS28 plays novel functions in cervical cancer progression; it is a novel prognostic marker and a potential therapeutic target.
